# Outcomes for Women with Gestational Diabetes Treated with Metformin: A Retrospective, Case-Control Study

**DOI:** 10.3390/jcm7030050

**Published:** 2018-03-09

**Authors:** Rachel T. McGrath, Sarah J. Glastras, Emma S. Scott, Samantha L. Hocking, Gregory R. Fulcher

**Affiliations:** 1 Department of Diabetes, Endocrinology and Metabolism, Level 3, Acute Services Building, Royal North Shore Hospital, St Leonards, Sydney NSW 2065, Australia; sarah.glastras@sydney.edu.au (S.J.G.); emmasigrids@bigpond.com (E.S.S.); samantha.hocking@sydney.edu.au (S.L.H.); greg.fulcher@sydney.edu.au (G.R.F.); 2 Northern Clinical School, University of Sydney, Sydney NSW 2065, Australia; 3 Kolling Institute of Medical Research, St Leonards, Sydney NSW 2065, Australia; 4 NHMRC Clinical Trials Centre, University of Sydney, Camperdown, Sydney NSW 2050, Australia; 5 Boden Institute, Charles Perkins Centre, University of Sydney, Camperdown, Sydney NSW 2006, Australia

**Keywords:** gestational diabetes, metformin, insulin, glycaemic control, perinatal outcomes

## Abstract

Metformin is increasingly being used a therapeutic option for the management of gestational diabetes mellitus (GDM). The aim of this study was to compare the maternal characteristics and perinatal outcomes of women with GDM treated with metformin (with or without supplemental insulin) with those receiving other management approaches. A retrospective, case-control study was carried out and 83 women taking metformin were matched 1:1 with women receiving insulin or diet and lifestyle modification alone. Women managed with diet and lifestyle modification had a significantly lower fasting plasma glucose (*p* < 0.001) and HbA1c (*p* < 0.01) at diagnosis of GDM. Furthermore, women managed with metformin had a higher early pregnancy body mass index (BMI) compared to those receiving insulin or diet and lifestyle modification (*p* < 0.001). There was no difference in mode of delivery, birth weight or incidence of large- or small-for-gestational-age neonates between groups. Women receiving glucose lowering therapies had a higher rate of neonatal hypoglycaemia (*p* < 0.05). The incidence of other adverse perinatal outcomes was similar between groups. Despite their greater BMI, women with metformin-treated GDM did not have an increased risk of adverse perinatal outcomes. Metformin is a useful alternative to insulin in the management of GDM.

## 1. Introduction

The incidence of gestational diabetes mellitus (GDM) is increasing in Australia and worldwide [1]. The latest diagnostic criteria recommended by the International Association of Diabetes and Pregnancy Study Groups (IADPSG) and endorsed by the Australasian Diabetes in Pregnancy Society (ADIPS) are more stringent than previous criteria and have led to an increase in the prevalence of GDM [2]. Following diagnosis, women with GDM routinely receive dietary education and lifestyle advice; with pharmacological therapy initiated if target blood glucose levels are not attained [3]. Importantly, treatment of GDM has been shown to significantly improve perinatal outcomes, with reductions in macrosomia, birth injury and neonatal death [4,5].

A number of studies have demonstrated that treatment of GDM with metformin can lead to adequate glycaemic control and does not increase the risk of adverse perinatal outcomes [6,7]. The mechanism of action of metformin comprises suppression of hepatic gluconeogenesis and increasing insulin sensitivity, which mitigates hyperglycaemia. Moreover, metformin may be a more favourable alternative to insulin as it is weight neutral, is not associated with hypoglycaemia, avoids the need for injections and may make follow-up simpler [8]. A recent clinical trial found that treatment with metformin from 12 to 18 weeks of gestation reduced gestational weight gain but had no effect on the development of GDM or neonatal birth weight in a population of obese women [9]. Furthermore, a large, prospective clinical trial that randomised women with GDM to insulin or metformin found that metformin therapy was not associated with an increased risk of perinatal complications [10]. In addition, several meta-analyses have found no difference in perinatal outcomes for women with GDM treated with metformin or insulin [11,12]. Whilst efficacious for the majority of women, some women will require additional therapy with insulin to achieve target blood glucose levels; however, the dose of insulin needed is usually less than those managed with insulin therapy alone [10].

Despite the above evidence, the use of metformin for the treatment of hyperglycaemia in pregnancy is not universally implemented and there is a lack of consensus in GDM management guidelines, dependent upon centre or location [13]. Moreover, a recent study found that metformin treatment in the first trimester of pre-gestational diabetes was associated with an increased risk of birth defects and pregnancy loss; however, these increased risks were attributed to hyperglycaemia rather than metformin therapy [14].

At our institution, the use of metformin for the treatment of GDM has been adopted as an alternative approach to insulin therapy in the management of hyperglycaemia in this population of women. Thus, the aim of the present study was to carry out an audit to assess and compare the maternal characteristics and perinatal outcomes of women with GDM treated with metformin (with or without supplemental insulin), in comparison to those treated solely with insulin or diet and lifestyle modification alone at our large tertiary referral hospital obstetric clinic in Sydney, Australia. This study will provide supplementary evidence of outcomes for women with GDM treated with metformin based upon our experience and the population of women that attended our institution. We hypothesise that our findings will support previous studies and show no increase in adverse perinatal outcomes for women treated with metformin in pregnancy.

## 2. Materials and Methods

### 2.1. Study Design

A retrospective, observational, case-control study was conducted through a review of the medical records of women with GDM in singleton pregnancy that attended the multi-disciplinary Specialist Obstetric Clinic (SOC) at Royal North Shore Hospital, Sydney, Australia, from September 2012 to August 2016. Approval for this study was obtained from the Northern Sydney Local Health District Human Research Ethics Committee (Study Reference No. RESP/15/107) and was carried out in keeping with the Strengthening the Reporting of Observational Studies in Epidemiology (STROBE) statement for case-control studies [15].

### 2.2. Diagnosis and Management of GDM

GDM was diagnosed using a 75-g oral glucose tolerance test (OGTT) with two diagnostic criteria used throughout the study period: a fasting plasma glucose of ≥5.5 mmol/L or a 2-h plasma glucose of ≥7.8 mmol/L until the end of 2014, with new diagnostic criteria of a fasting plasma glucose ≥5.1 mmol/L, a 1-hour plasma glucose ≥10 mmol/L or a 2-h plasma glucose ≥8.5 mmol/L introduced in 2015, as endorsed by ADIPS [16]. At diagnosis of GDM, a diabetes nurse educator and dietician provided all women with group diabetes education and dietary advice. Dietary advice comprised information about a carbohydrate modified diet (30–45 g carbohydrate at main meals, 15–30 g carbohydrate at mid meals and encouragement to eat low glycaemic index carbohydrate). In addition, women were instructed to monitor their blood glucose levels 4 times daily using a blood glucose meter: fasting and 2-hs post breakfast, lunch and dinner. The standard model of care for women with GDM is to attend the SOC for review by an endocrinologist, and management was instituted dependent upon target blood glucose levels of <5.5 mmol/L fasting until 2014, ≤5.0 mmol/L fasting from 2015 and <6.7 mmol/L 2-h postprandially. If target blood glucose levels were not achieved with lifestyle and diet modification alone, pharmacological therapy was initiated. The use of metformin was dependent upon physician and patient preference. Further, if women failed to achieve adequate glycaemic control with metformin, treatment was intensified by the addition of insulin. Women managed with diet and lifestyle modification alone attended a midwife and diabetes educator-led diabetes education group session every 1–4 weeks.

The metformin group comprised women with GDM that were prescribed metformin as first-line therapy with/without the addition of insulin. Metformin was initiated at a dose of 500 mg twice daily and up-titrated to 2 g per day (where tolerated) if adequate glycaemic control was not achieved. Supplemental insulin was introduced if metformin was not sufficient to ameliorate hyperglycaemia. Women in the insulin-alone group were those who were prescribed insulin as their initial glucose lowering therapy. Insulin was commenced (Protaphane or Levemir and/or NovoRapid) depending upon the pattern of hyperglycaemia and the dose increased to achieve target fasting and postprandial blood glucose levels. Diet and lifestyle modification comprised non-pharmacological treatment as outlined above. Patients in the insulin and diet and lifestyle modification groups were individually matched in a 1:1 ratio to patients in the metformin group for age, previous history of GDM and gestational age at diagnosis of GDM.

### 2.3. Data Collection

Data extracted from patient electronic medical records included maternal demographics (age, ethnicity, body mass index (BMI) recorded at the first visit to the antenatal clinic, family history of diabetes, previous history of GDM), gestational age at diagnosis of GDM, treatment regimen and the following perinatal outcomes: maternal outcomes—mode and timing of delivery, and neonatal outcomes—birth weight, gestational age at delivery, large-for-gestational-age (LGA; defined as birth weight >90th centile for gestational age and gender) [17], small-for-gestational-age (SGA; defined as birth weight <10th centile for gestational age and gender) [17], shoulder dystocia, respiratory distress, neonatal hypoglycaemia, jaundice, neonatal intensive care unit (NICU) admission, birth injury and neonatal death.

### 2.4. Statistical Analysis

To compare differences in birth weight between women treated with metformin or insulin and based upon the results of Rowan et al. [10], a sample size of 3045 per group would be required. However, to detect difference in fasting blood glucose levels in women treated with metformin compared with insulin or diet alone (a secondary outcome of this study) and using the results of Corbould et al. [18], with power of 0.80 and α of 0.05, a sample size of 23 per group is needed.

Differences between groups were compared using Student’s independent *t*-test and one-way analysis of variance (ANOVA) for continuous data and chi-squared test and logistic regression for categorical data. Statistical analyses were carried out using GraphPad Prism Version 6 (GraphPad Software, La Jolla, CA, USA) and IBM SPSS Version 22 (IBM, Armonk, NY, USA). A *p* value of <0.05 was considered statistically significant.

## 3. Results

### 3.1. Study Population and Demographics

During the time period of September 2012 to April 2016, 86 women were identified as taking metformin (with or without supplemental insulin) during singleton GDM pregnancy. Three of these women were subsequently excluded from the analysis as they were taking insulin prior to metformin. Thus, 83 women taking metformin were matched to those taking insulin or management with diet and lifestyle modification alone. The demographics of the groups are outlined in Table 1.

Women treated with metformin had a higher early pregnancy BMI compared to the insulin and diet and lifestyle-modification groups (*p* < 0.001). There was no difference in family history of diabetes between groups.

### 3.2. Glycaemic Control

**Figure 1:**
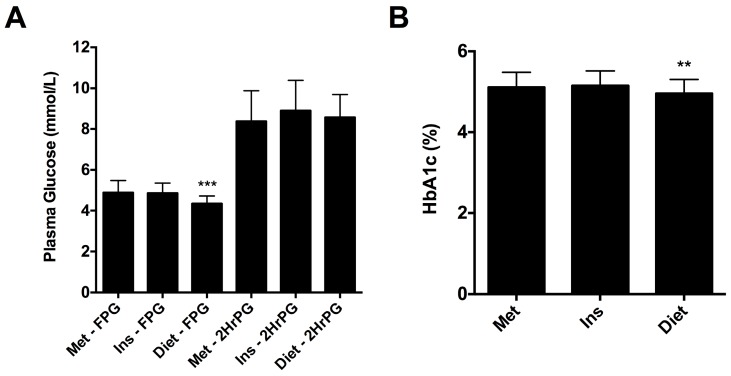
(**A**) Fasting plasma glucose (FPG) and 2-h plasma glucose post 75 g oral glucose tolerance test (OGTT) (2HrPG) for women diagnosed with GDM that were subsequently managed with metformin (Met), insulin (Ins) or diet and lifestyle modification (Diet). (**B**) HbA1c at diagnosis of GDM for women subsequently treated with metformin, insulin or diet and lifestyle modification. Results expressed as mean ± standard deviation. ** *p* < 0.01; *** *p* < 0.0001.

Women that were successfully managed with diet and lifestyle modification alone had a significantly lower fasting plasma glucose level on 75 g OGTT and HbA1c at diagnosis of GDM compared to women receiving metformin or insulin therapy (4.4 mmol/L vs. 4.9 mmol/L vs. 4.8 mmol/L, respectively; *p* < 0.0001 (Figure 1A) and 5.0% vs. 5.1% vs. 5.2%, respectively; *p* < 0.01 (Figure 1B)). There was no difference in the gestational age at which pharmacotherapy was initiated between the metformin and insulin groups (Table 1). In addition, the proportion of women taking metformin that required additional therapy with insulin to achieve targets was 42.2% (*n* = 35) and the mean gestational age at which insulin was added was 29.5 ± 5.7 weeks. There was no difference in the total daily dose of insulin at delivery for women in the metformin (and supplemental insulin) and insulin groups.

### 3.3. Perinatal Outcomes

The maternal and neonatal outcomes for women taking metformin (with or without additional insulin) versus those taking insulin alone or managed with diet and lifestyle modification are outlined in Table 2. There was no difference in the rate of normal vaginal delivery, instrumental delivery or Caesarean section between groups. Women treated with glucose lowering therapies were delivered at a slightly earlier gestational age than women managed with lifestyle measures (*p* = 0.008). Furthermore, there was no difference in birth weight and a comparable incidence of SGA and LGA neonates was observed in each group. Following adjustment for early-pregnancy BMI, there was no association between GDM treatment and birth weight centile (*p* = 0.437) yet women managed with diet and lifestyle modification alone were less likely to have LGA neonates compared with women receiving pharmacotherapy (OR: 0.58; 95% CI: 0.358 to 0.940; *p* = 0.027). There was no difference in the likelihood of women managed with metformin (with or without supplemental insulin) or insulin having LGA neonates (OR: 0.548; 95% CI: 0.237 to 1.266; *p* = 0.159).

The rate of adverse perinatal outcomes was similar between groups. Infants of women treated with glucose-lowering therapies were more likely to experience neonatal hypoglycaemia (*p* = 0.039; Table 2). Of note, almost two thirds (61.5%) of the cases of neonatal hypoglycaemia in the metformin group were in women taking insulin in addition to metformin. For women treated with metformin alone, there was no difference in the rate of neonatal hypoglycaemia in comparison to the diet and lifestyle-modification group (10.4% vs. 6.1%; *p* = 0.497). There was also no difference in the incidence of shoulder dystocia, respiratory distress, jaundice, NICU admission, birth injury, birth defect or neonatal death in women treated with metformin compared to those managed with insulin or diet and lifestyle modification. The birth defects observed in each of the groups were tongue tie (metformin, *n* = 3; insulin, *n* = 1; diet and lifestyle modification, *n* = 3), left-lung sequestration (diet and lifestyle modification, *n* = 1), hypospadias (insulin, *n* = 1) and bilateral talipes (metformin, *n* = 1).

## 4. Discussion

The results of the present analysis demonstrate that treatment of women with GDM with metformin gives rise to similar perinatal outcomes in comparison to women managed with insulin alone or diet and lifestyle modification alone. No difference was observed in the incidence of neonatal complications between groups, including respiratory distress, jaundice or birth defects; however, women treated with metformin and supplemental insulin or insulin alone were more likely to have infants experiencing neonatal hypoglycaemia, compared with those managed with lifestyle therapy alone. Lower fasting plasma glucose levels and HbA1c at diagnosis of GDM were associated with successful management without pharmacotherapy. Furthermore, women requiring glucose-lowering therapy were more likely to have LGA neonates than those managed with diet and lifestyle modification alone, indicating that hyperglycaemia requiring treatment is associated with an increased incidence of LGA neonates. In addition, women with a greater early-pregnancy BMI were more likely to be prescribed metformin therapy, which we speculate may be due to the beneficial effects of metformin on insulin resistance or the desire to avoid weight gain that may be incurred with insulin treatment.

The majority of guidelines recommend insulin for the treatment of hyperglycaemia in GDM [16,19]; however, there are several barriers towards insulin use in this population, namely an undesirable route of administration, the potential for weight gain (which may further compound and propagate hyperglycaemia) and the risk of hypoglycaemia [20]. Thus, metformin may be a more acceptable therapy for women with GDM. Studies have demonstrated that metformin is safe for use in pregnancy, with no significant differences in adverse neonatal outcomes such as respiratory distress and NICU admission [21]. The incidence of perinatal complications in the present study population are similar to that observed by Spaulonci et al. [22] and Goh et al. [23]. Treatment with metformin did not give rise to a reduction in prematurity, jaundice or NICU admission, as has been shown in previous reports [24]. This difference may result from the smaller sample size used. Alternatively, the absolute rate of adverse perinatal outcomes in our population was comparatively low and this may reflect optimal obstetric management, regardless of the therapy used to manage glycaemia in GDM.

Women prescribed metformin had a significantly higher early pregnancy BMI than the other groups suggesting that physicians may believe that metformin will be of most benefit to women that are overweight or obese who have a higher likelihood of significant insulin resistance. It has recently been shown that maternal weight gain in GDM is an independent predictor of macrosomia [25,26]. Therefore, optimisation of maternal weight prior to or early in pregnancy may avoid the need for pharmacologic interventions and improve perinatal outcomes, and the weight-neutral properties of metformin may be considered favourable for women with an elevated BMI. In addition, increased BMI in pregnancy has been shown to be associated with large-for-gestational-age neonates and maternal complications [27,28]; however, women in the metformin group did not display an increase in the incidence of adverse perinatal outcomes. A limitation of the present study is that we were unable to assess gestational weight gain, which has previously been shown to be a favourable outcome for women treated with metformin in pregnancy [11].

In women with mild GDM, metformin therapy alone is sufficient to achieve glycaemic targets [17,18]. In our population, just under half of the women treated with metformin required additional therapy with insulin for glycaemic control. This is a similar finding to other studies, wherein a significant proportion of women will not achieve glycaemic targets with metformin monotherapy alone [10,18]. We have recently observed that women with GDM treated with metformin who required pharmacological intervention earlier in pregnancy were more likely to need supplemental insulin as the pregnancy progressed [29].

A recent meta-analysis demonstrated that use of metformin in pregnancy is not associated with an increased risk of short-term adverse outcomes and may have benefit in the postnatal period [30]. Furthermore, children of women exposed to metformin during GDM pregnancy were compared to those exposed to insulin at 6, 12 and 18 months of age and no difference was observed in early motor and linguistic skills and social development, although children in the metformin group were heavier [31]. In addition, the Metformin in Gestational Diabetes: The Offsrping Follow-Up (Mig-TOFU) study found similar outcomes for children of mothers treated with metformin in pregnancy [32,33]. It has been hypothesised that exposure to metformin in utero may be of benefit to children as it may increase insulin sensitivity from a young age and reduce metaboliccomplications [24]; however, longer term studies are required to confirm this assumption and to ensure the safety of metformin for neurodevelopment in children.

A limitation of this study is the small sample size and the lack of statistical power for comparison of adverse perinatal outcome rates between groups; however, we tried to account for this by using well-matched controls to limit the potential for confounding and spurious results. It is possible that with a larger sample size, differences in perinatal outcomes may have been observed between groups, yet our results are in keeping with other studies that have examined outcomes post exposure to metformin in pregnancy [34]. The majority of studies examining metformin therapy in GDM have compared outcomes to women taking insulin, whereas we have employed an additional control group comprising women managed with diet and lifestyle modification alone. Both Goh et al. [23] and Corbould et al. [18] utilised a similar methodological approach and found no difference in neonatal outcomes between women treated solely with metformin, insulin or those who were diet-controlled. An additional limitation is that this was not a randomised study and therefore, metformin use was due to patient and/or physician preference.

In summary, treatment of GDM with metformin (with or without supplemental insulin) gives rise to similar perinatal outcomes to women managed solely with insulin or diet and lifestyle modification. Metformin is a useful alternative to insulin in the management of GDM, particularly in women with an elevated BMI. Our results also demonstrate that the use of metformin in an Australian GDM cohort has similar outcomes to other populations. With the results of long-term outcome studies examining the impact of metformin exposure in utero expected to be published in the near future, it is anticipated that the acceptance of metformin for the treatment of GDM will be further increased.

## Figures and Tables

**Table 1:** Demographics and characteristics of women with gestational diabetes mellitus (GDM) managed with metformin, insulin or diet and lifestyle modification.

Maternal Characteristics	Metformin (*n* = 83)	Insulin (*n* = 83)	Diet and Lifestyle (*n* = 83)	*p*
Maternal age (years)	33.1 ± 4.8	33.5 ± 4.3	33.1 ± 4.3	0.858
Ethnicity				
Caucasian	41.0% (*n* = 34)	30.1% (*n* = 25)	37.3% (*n* = 31)	0.261
South Asian	26.5% (*n* = 22)	34.9% (*n* = 29)	18.1% (*n* = 15)	-
Asian	21.7% (*n* = 18)	24.1% (*n* = 20)	32.5% (*n* = 27)	-
South-East Asian	7.2% (*n* = 6)	6.0% (*n* = 5)	9.6% (*n* = 8)	-
Middle Eastern	2.4% (*n* = 2)	4.8% (*n* = 4)	1.2% (*n* = 1)	-
Pacific Islander	1.2% (*n* = 1)	0	0	-
African	0	0	1.2% (*n* = 1)	-
Previous GDM	13.3% (*n* = 11)	13.3% (*n* = 11)	13.3% (*n* = 11)	0.999
Diagnosis of GDM (weeks)	23.6 ± 5.9	24.0 ± 5.8	24.0 ± 5.7	0.880
Maternal BMI (kg/m^2^)	27.8 ± 8.0	25.2 ± 6.3	22.7 ± 2.9	<0.0001
Family history of diabetes	42.2% (*n* = 35)	48.2% (*n* = 40)	39.8% (*n* = 33)	0.528
Gestational age at initiation of pharmacotherapy (weeks)	27.1 ± 5.7	28.0 ± 5.4	N/A	0.292
Gestational age at initiation of supplemental insulin (weeks)	29.5 ± 5.7	N/A	N/A	-
Total daily dose of insulin in units (before delivery)	23.6 ± 19.6	26.9 ± 24.2	N/A	0.779

**Table 2:** Maternal and neonatal outcomes for women receiving metformin in comparison to insulin or diet and lifestyle modification.

Perinatal Outcomes	Metformin (*n* = 83)	Insulin (*n* = 83)	Diet and Lifestyle (*n* = 82)	*p*
**Maternal Outcomes**				
Mode of Delivery				
Vaginal	38.6% (*n* = 32)	50.6% (*n* = 42)	53.7% (*n* = 44)	0.268
Instrumental	19.3% (*n* = 16)	19.3% (*n* = 16)	13.4% (*n* = 11)	-
Caesarean section	42.2% (*n* = 35)	30.1% (*n* = 25)	32.9% (*n* = 27)	-
**Neonatal Outcomes**				
Birth weight (g)	3289 ± 503	3150 ± 497	3228 ± 511	0.140
Birth weight centile	61 ± 28.5	52.8 ± 27.1	53.7 ± 26.6	0.089
Large-for-gestational-age	21.7% (*n* = 18)	14.5% (*n* = 12)	8.5% (*n* = 7)	0.059
Small-for-gestational-age	4.1% (*n* = 3)	4.1% (*n* = 3)	5.6% (*n* = 4)	0.758
Gestational age at delivery (weeks)	38.6 ± 1.2	38.4 ± 1.5	38.9 ± 1.7	0.008
Shoulder dystocia	4.1% (*n* = 4)	5.5% (*n* = 4)	4.2% (*n* = 3)	0.909
Respiratory distress	6.8% (*n* = 5)	11.0% (*n* = 8)	13.9% (*n* = 10)	0.389
Neonatal hypoglycaemia	15.7% (*n* = 13)	19.3% (*n* = 16)	6.1% (*n* = 5)	0.039
Jaundice	21.7% (*n* = 18)	18.1% (*n* = 15)	14.6% (*n* = 12)	0.501
NICU admission	14.5% (*n* = 12)	14.5% (*n* = 12)	7.3% (*n* = 6)	0.268
Birth injury	0	0	0	-
Birth defect	4.8% (*n* = 4)	2.4% (*n* = 2)	4.9% (*n* = 4)	0.645
Neonatal death	0	0	1.2% (*n* = 1)	-
